# Latest Advances in Image Acceleration: All Dimensions are Fair Game

**DOI:** 10.1002/jmri.28462

**Published:** 2022-10-07

**Authors:** Camila Munoz, Anastasia Fotaki, René M. Botnar, Claudia Prieto

**Affiliations:** ^1^ School of Biomedical Engineering and Imaging Sciences King's College London London UK; ^2^ Escuela de Ingeniería Pontificia Universidad Católica de Chile Santiago Chile; ^3^ Millenium Institute for Intelligent Healthcare Engineering iHEALTH Santiago Chile

**Keywords:** image acceleration, multidimensional MRI, cardiac MR, abdominal MR

## Abstract

Magnetic resonance imaging (MRI) is a versatile modality that can generate high‐resolution images with a variety of tissue contrasts. However, MRI is a slow technique and requires long acquisition times, which increase with higher temporal and spatial resolution and/or when multiple contrasts and large volumetric coverage is required. In order to speedup MR data acquisition, several approaches have been introduced in the literature. Most of these techniques acquire less data than required and exploit intrinsic redundancies in the MR images to recover the information that was not sampled. This article presents a review of MR acquisition and reconstruction methods that have exploited redundancies in the temporal, spatial, and contrast/parametric dimensions to accelerate image data acquisition, focusing on cardiac and abdominal MR imaging applications. The review describes how each of these dimensions has been separately exploited for speeding up MR acquisition to then discuss more advanced techniques where multiple dimensions are exploited together for further reducing scan times. Finally, future directions for multidimensional image acceleration and remaining technical challenges are discussed.

**Evidence Level:**

5

**Technical Efficacy:**

1

Accelerating image data acquisition has been a sustained goal in magnetic resonance imaging (MRI). In a variety of clinical applications, particularly cardiac, thoracic, and abdominal imaging, faster data acquisition is key to enable higher spatial resolution and volumetric coverage and to capture changes in signal intensity with sufficient temporal resolution while maintaining clinically feasible scan times. Furthermore, reducing scan times can improve patient comfort and minimize the impact of physiological motion, which can cause artifacts in the images.

In conventional MRI, the prescribed field of view (FOV) and spatial resolution of the images define the amount of information that needs to be collected in *k*‐space to fulfil the Nyquist criterion and produce good‐quality images. Several intrinsic factors impact the duration of a MR scan, including required tissue contrast, signal‐to‐noise ratio, and underlying properties of the tissue types involved. For a given imaging application, the amount of *k*‐space information required (usually expressed as a number of *k*‐space points or readouts) will then define the total duration of the scan. Conversely, for imaging applications where breath‐holding or real‐time imaging is required to minimize the adverse effects of physiological motion in image quality, the limited scan time imposes a boundary on spatial resolution and volumetric coverage.

A straightforward option to reduce data acquisition time is to collect fewer than the required number of *k*‐space samples, that is, to *undersample k*‐space. Over the last decades, a large variety of data acquisition and image reconstruction techniques have been proposed in the literature to produce images of acceptable quality from undersampled data. One of the most used techniques for image acceleration in clinical practice is parallel imaging.^1^ Parallel imaging approaches use arrays of receiver coils to obtain multiple measurements from the object of interest and exploit this additional information to reconstruct good‐quality images. These techniques are robust and have been integrated into most of clinical MR scanners, achieving two to four times reduction in scan time for most applications. To further accelerate data acquisition, research efforts have additionally explored exploiting intrinsic redundancies in the MR images and/or in *k*‐space to fully or partially recover the information that was not sampled. For instance, some of these methods have exploited spatial redundancies, by using the fact that medical images are often compressible with little or no loss of information, and that neighboring areas within an image tend to have similar signal characteristics.^2^ These methods can be said to exploit redundancies in the *spatial dimension*.

Other approaches have focused on exploiting redundancies between successive images in applications where time series are obtained, by assuming that images close in time are mostly similar, with just a few voxels changing in intensity between them. These methods therefore exploit redundancies in the *temporal dimension*.^3^ This type of redundancy has been exploited in applications such as cardiac cine imaging, where the aim is to capture information about the motion of the heart, and in dynamic contrast‐enhanced (DCE) MRI, where the aim is to capture the changes in contrast agent concentration in different tissues to provide information about the anatomy and function.

Depending on the type of MR imaging being performed, additional dimensions might be available. For example, in conventional quantitative parametric mapping, usually a few (about 5–10) images with different contrasts are acquired and subsequently fitted to a model to retrieve the parameter of interest.^4^, ^5^ Similar to the case of DCE MRI, because all the contrast images relate to the same anatomical object, there is redundant information between them that can be exploited for image acceleration. Furthermore, the signal behavior for a tissue with a given set of parameters can be predicted by using a physical model of the pulse sequence employed to acquire the data; such models can be used to create dictionaries of expected signal evolutions, with a number of entries typically smaller than the number of pixels in the images,^6^ indicating that there are redundancies in the *relaxation/parametric dimension*.

While each of these dimensions can be exploited separately, further acceleration can be achieved by exploiting multiple dimensions at a time. Indeed, some early developments in image acceleration exploited both spatial and temporal dimensions in 2D cardiac cine imaging applications, achieving acceleration factors of up to 4–6×.^7^, ^8^ During the last two decades, advances in sequence development and *k*‐space trajectory design, in combination with novel undersampled image reconstruction techniques have enabled the acquisition of higher dimensional datasets, where now volumetric 3D spatial redundancies (instead of the conventional 2D ones), with multiple contrast (eg T1, T2 recovery) and temporal dynamics (cardiac, respiratory, etc) can be resolved or exploited to enable higher acceleration factors for multidimensional imaging within clinically feasible scan times.^9^, ^10^ These approaches rely on 4D (eg 3D spatial + time), 5D (eg 3D + cardiac dynamics + respiratory dynamics) or even 6D (eg 3D + cardiac dynamics + respiratory dynamics + contrast dynamics) image reconstruction techniques. Figure 1 shows one example of such 6D datasets for free‐breathing multicontrast cardiac imaging obtained with a joint T1/T1*ρ* mapping sequence,^9^, ^10^ with three spatial dimensions, a contrast dimension to enable T1 and T1*ρ* mapping, a respiratory dimension, and a multiple‐echo dimension to enable water/fat imaging.^11^ This type of multidimensional imaging may offer additional information that can be used for a comprehensive assessment of disease from integrated efficient single‐scan examinations.

**FIGURE 1:**
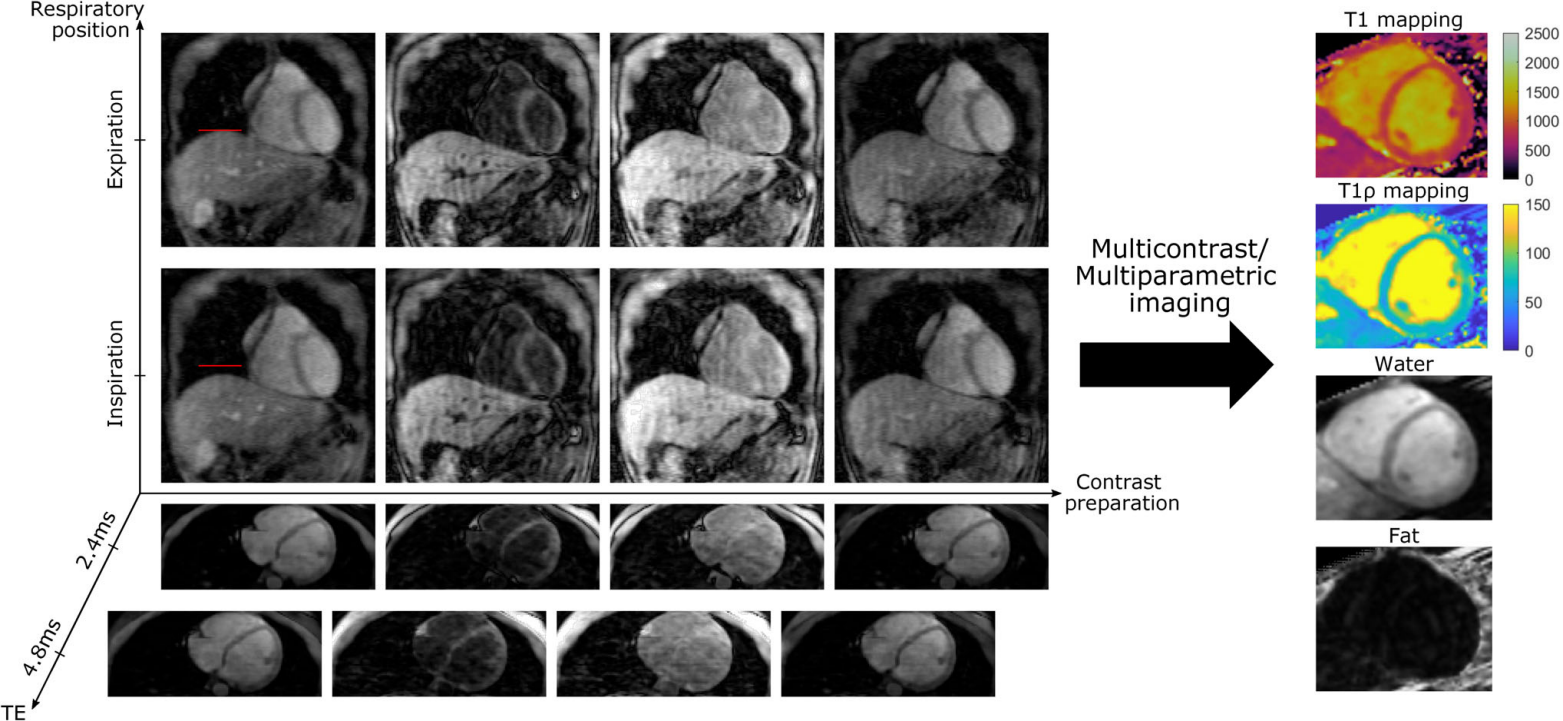
Example 6D cardiac imaging obtained for multicontrast multiparametric mapping, including three spatial dimensions, a contrast preparation dimension for T1 and T1*ρ* mapping, a respiratory dimension (showing inspiration and expiration respiratory states), and echo times (TE) for water/fat imaging.

This article presents a historical review of MR acquisition and reconstruction methods that have exploited these dimensions to accelerate image data acquisition in MRI. Due to space constraints, this review will not describe parallel imaging techniques and will mainly focus on cardiac and abdominal imaging applications, covering only a subset of the available techniques that have been introduced in the literature. We will attempt to provide a conceptual understanding of how the different dimensions can be exploited for image acceleration, without going into details about the technical aspects of each method, which can be found in the corresponding references.

The rest of this article is divided as follows. First, we will introduce the key concepts that underlie undersampled MR image reconstruction. We then will explore the techniques that have been used to accelerate image acquisition by exploiting redundancies in one dimension at a time, before moving into describing multidimensional methods.

## MR Reconstruction: A Regularized Inverse Problem

**FIGURE 2:**
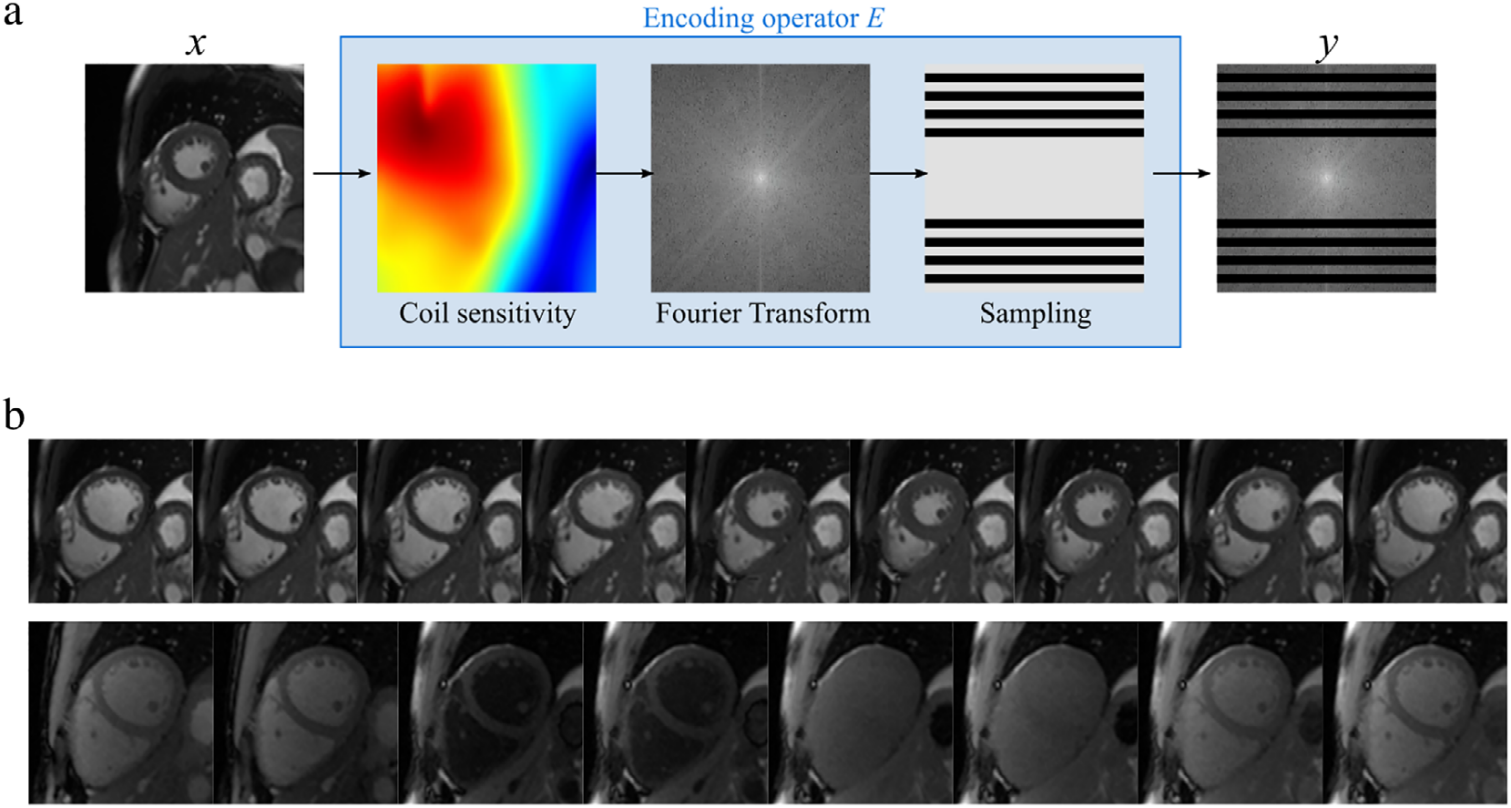
(a) MR measurement (i.e. forward) model, where x is the object being scanned, E is the MR encoding operator, including coil sensitivity profiles, Fourier transform and *k*‐space sampling mask, and y is the measured k‐space data. (b) Example MR images where x represents a multidimensional object, for example, a series of cardiac cine images (top), or a set of T_1_‐weighted images for cardiac T_1_ mapping (bottom).

MR image reconstruction can be formulated as an inverse problem, because we aim to reconstruct the unknown representation of the object (i.e. an image) from the sampled measurements in *k*‐space. In general, the equation that describes the data acquisition in MR (i.e. the forward model) can be expressed as y=Ex+ε, where x is the unknown image to be reconstructed, E is the MR encoding operator, including coil sensitivity profiles, Fourier transform and k‐space sampling mask, y is the measured *k*‐space data, and ε is the measurement noise (Fig. 2a).

The MR image reconstruction problem consists therefore of recovering the image x from the known encoding operator E and the acquired data y. When undersampling is used to accelerate data acquisition, this inverse problem becomes ill‐posed, and its solutions can become unstable. To overcome this issue, additional information about x can be used to stabilize the solution, with the reconstruction problem re‐formulated as

(1) $$\underset{x}{\text{argmin}}{\left\|\mathrm{Ex}-y\right\|}_{2}^{2}+\mathrm{\lambda R}\left(x\right)$$

where $R\left(x\right)$ is a regularization term that enforces some known property of x, and λ is the regularization parameter that controls the trade‐off between the consistency with the acquired data (${\left\|\mathrm{Ex}-y\right\|}_{2}^{2}$ term) and the regularization term ($R\left(x\right)$). In other words, we attempt to find the optimal image x that is consistent with the acquired undersampled data and at the same time satisfies some known property or prior information that we have about the image to be reconstructed.

In its simplest form, this model represents the acquisition of a single image; however, it can be straightforwardly extended to include multiple images. For example, x can represent a series of cardiac cine images (Fig. 2b, top), or a set of T_1_‐weighted images for T_1_ mapping (Fig. 2b, bottom). The redundant information present in the different dimensions of x can be exploited in such cases as prior information to stabilize the solution through an appropriate choice of the regularization function $R\left(x\right)$.

Two main ideas have been widely used in the undersampled image reconstruction literature to aid the choice of $R\left(x\right)$. The first of these notions is that MR images have a *sparse representation* in some transformed domain: this means that there is a mathematical operation that can convert the image x into a domain where most coefficients of its basis are zero or nearly zero. Therefore, a reduced number of coefficients (much fewer than the number of pixels/voxels in the original image) is sufficient to comprise all the information contained in the image. Compressed‐sensing^2^, ^12^ methods take advantage of this by solving the following problem:

(2) $$\underset{x}{\text{argmin}}{\left\|\mathrm{Ex}-y\right\|}_{2}^{2}+\lambda {\left\|\Psi x\right\|}_{1}$$

where the L_1_ norm (${\left\|\cdot \right\|}_{1}$) enforces sparsity in the transformed domain Ψx, with Ψ known as the sparsifying transform.

The second idea is the *low rankness* of MR images when represented as a matrix or a tensor.^13^ The rank of a matrix represents its number of uncorrelated rows or columns and thus represents the amount of nonredundant information present contained in such matrix. A matrix is said to be low rank when it has only a few large singular values and therefore can be fully represented by a reduced number of singular vectors and values, in a similar fashion to the idea of sparse representation described above. The reconstruction problem can then be formulated as

(3) $$\underset{x}{\text{argmin}}{\left\|\mathrm{Ex}-y\right\|}_{2}^{2}$$

(4) $$\text{such that}\;\mathrm{rank}\left(x\right)\leq R$$

In order to reconstruct the images, algorithms proposed in the literature have implicitly enforced low rankness by using the Schatten p‐norm (p<1) or the nuclear norm (p=1) as a regularization term, with the Schatten norm of a matrix x defined as

(5) $${\left\|x\right\|}_{p}={\left(\sum_{i}\sigma_{i}^{p}\right)}^{1/p}$$

with $\sigma_{i}$ the singular values of x, usually obtained by means of a singular value decomposition (SVD). Alternatively, some approaches have used an explicit formulation to enforce low rankness. These include the SVD, which involves a matrix decomposition that allows the preservation of the relevant singular values required for representing the image and discards redundant values, without compromising image quality. Similarly, principal component analysis (PCA) acts as variation reduction algorithm that transforms a set of correlated variables into as smaller set of uncorrelated variables (the principal components) that retain the significant information of the original data. In order to extend this idea to multidimensional MR datasets, tensor formulations have been introduced, whereby arranging the data into higher‐dimensional tensors, low rankness can be enforced using higher‐order SVD and/or tensor decomposition.

Both sparsity and low‐rank ideas have been used separately or together throughout the development of image acceleration techniques, and we will often come back to them in this review article.

## Exploiting Temporal Redundancies

Early methods proposed for accelerating dynamic MRI took advantage of the similarity between neighboring time frames to reduce the amount of data acquired at each time frame. Indeed, view‐sharing (variously known also as sliding window, moving average reconstruction) techniques have been around since the late 1980s.^14^, ^15^, ^16^ These techniques use different undersampling patterns in *k*‐space at each time frame, so that the full *k*‐space is successively update at a certain rate. The missing *k*‐space samples are subsequently filled with the closest data point available from neighboring frames. While this approach can produce images with seemingly good quality, there is loss of temporal fidelity and edges from fast moving objects may become blurred. Nevertheless, due to its simplicity and robustness, this approach has been used in several clinical applications, particularly for time‐resolved MR angiography^17^, ^18^, ^19^ and cardiac cine imaging.^20^, ^21^

It is worth noting that in some of these applications, the temporal dimension does not represent actual time, but the relative position of each time frame within the cardiac and/or the breathing cycles. In these applications, the acquired *k*‐space data can be assigned to one of several bins or phases using an underlying assumption of periodicity or quasi‐periodicity of the cardiac and breathing cycles. Each of these phases therefore contains data acquired at a similar position over multiple cardiac and/or breathing cycles, with minimal interbin motion. In subjects with regular respiratory and cardiac cycles, this assumption of periodicity can result in good‐quality images, particularly for acquisitions with short scan times. However, in longer scans, breathing patterns can drift significantly, and some subjects might present with cardiac arrhythmias and heart rate variability, negatively affecting the quality of resulting images.

In the early techniques mentioned above, each of these phases was reconstructed independently after sharing *k*‐space data. More recently, the development of compressed‐sensing techniques opened new possibilities for the simultaneous reconstruction of time‐resolved images. The *k*‐*t* SPARSE method^22^ pioneered the application of compressed sensing for dynamic MRI, using a one‐dimensional Fourier transform (FT) across the temporal dimension as sparsifying transform for 2D cardiac cine imaging. Similar works followed, applying temporal FT to accelerate carotid flow imaging up to a factor of 4×.^23^

In order to further reduce scan time, *k*‐*t* SPARSE SENSE combined sparsity in the temporal dimension and parallel imaging, achieving a factor of up to 8× acceleration in first‐pass cardiac perfusion imaging.^24^ This approach was subsequently extended to real‐time cardiac cine^25^ and phase‐contrast imaging,^26^ using either temporal FT, temporal finite differences (also known as temporal total variation [TV]), temporal principal component analysis (PCA) or a combination of them as sparsifying transform. GRASP^27^ is an extension of k‐t SPARSE SENSE to the golden angle radial trajectory and uses temporal TV as regularization term, which encourages the difference between frames to be sparse. Because of the use of a golden angle radial trajectory, GRASP has some robustness against respiratory motion and has been applied successfully to free‐breathing abdominal imaging.^27^, ^28^, ^29^

**FIGURE 3:**
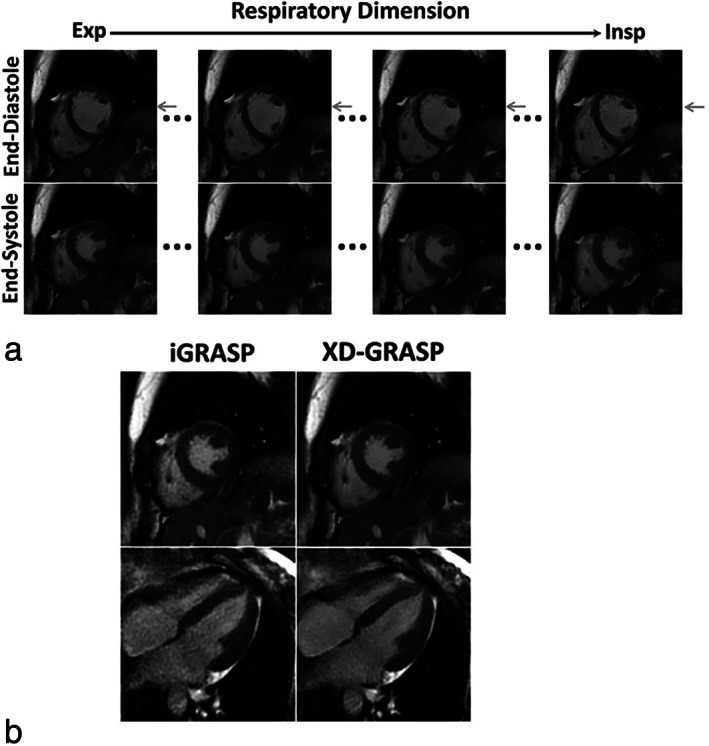
Images obtained with the XD‐GRASP approach for free‐breathing 2D cardiac CINE imaging, with sparsity exploited in both the respiratory and cardiac dimensions. (a) XD‐GRASP provides cardiac and respiratory‐resolved images (gray arrows indicate different respiratory motion states). (b) Exploiting sparsity along two dynamic dimensions (XD‐GRASP, right‐hand column) improves image quality compared to exploiting sparsity along a single dynamic dimension only (iGRASP, left‐hand column). *Source*: Reproduced with permission from reference 30

However, in patients with more extensive breathing patterns, this approach may result in blurring and artifacts. To address this issue, Feng et al introduced the XD‐GRASP (eXtra‐Dimensional GRASP) approach,^30^ where the acquired dataset is sorted into bins according to more than one temporal dimension (eg cardiac phases, respiratory phases, contrast enhancement), and additional sparsifying transformations are added to the reconstruction problem. XD‐GRASP was first demonstrated for free‐breathing 2D cardiac cine imaging, where authors used temporal TV along the cardiac and the respiratory dimensions to reconstruct good‐quality cardiac‐ and respiratory‐resolved images. Authors showed that simultaneously exploiting sparsity in more than one dynamic dimension improves image quality (Fig. 3). The XD‐GRASP method has since been used in a variety of clinical applications, including liver imaging,^31^ coronary MR angiography,^32^ and cardiac‐ and respiratory‐resolved 5D whole heart imaging,^33^ among others. Although in some of these applications, the motion‐resolved approach is used to improve image quality, and not necessarily to reduce overall scan time, the same framework can be used to speed up the acquisition. For instance, the XD‐GRASP approach is 5× accelerated for efficient aortic MR angiography in the literature,^34^ while a 3.3× acceleration is used in the literature^35^ for 3D late gadolinium enhancement (LGE) imaging of the left atrial wall. Finally, while the XD‐GRASP method was originally proposed for radial k‐space trajectories, extensions have been proposed for Cartesian imaging in the literature.^36^, ^37^

## Exploiting Spatial Redundancies

Early developments in MR image acceleration aimed to exploit redundancies present in the image domain or in *k*‐space, including partial Fourier and reduced FOV approaches.^38^ Following the introduction of phased‐array coils, additional information arising from the local sensitivity of the different elements in the coils enabled the development of parallel imaging techniques,^39^, ^40^, ^41^ which were very successful in reducing scan time by factors of 2–4× for most applications and are still widely used in clinical MR examinations.^1^

Similar to the case of temporal redundancies, the introduction of compressed‐sensing enabled increased undersampling factors for further image acceleration by providing a new framework for exploiting spatial redundancies in MR images. Furthermore, this increased acceleration factors enabled the acquisition of volumetric 3D datasets with sufficient spatial resolution for several clinical applications, which otherwise would have been prohibitively long. A comprehensive review of compressed‐sensing techniques is out of the scope of this manuscript and can be found elsewhere.^42^, ^43^, ^44^ We will however briefly review some of the different ways that spatial redundancies can be exploited for image acceleration.

**FIGURE 4:**
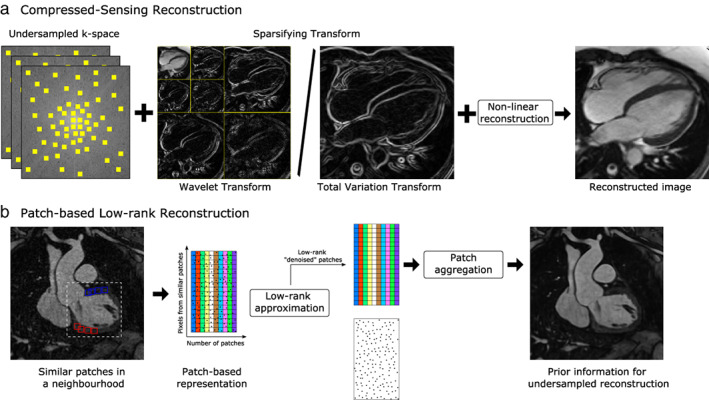
Schematic diagram of (a) compressed‐sensing reconstruction and (b) patch‐based low‐rank reconstruction for undersampled MR imaging. (a) Compressed‐sensing reconstruction requires a sampling pattern that produces incoherent artifacts, a sparsifying transform such as wavelet or total variation, and a nonlinear reconstruction algorithm, to produce artifact‐free images from undersampled data. (b) Patch‐based low‐rank methods search for similar patches within neighborhoods in the image and use a low‐rank approximation to remove noise‐like artifacts. After patches are aggregated, the denoised volume can be used as prior knowledge to regularize the undersampled image reconstruction problem.

Most medical images have sparse representations in the finite differences domain, wavelet domain, or the discrete cosine transform domain (Fig. 4, top),^2^ offering a variety of potential sparsifying transforms that have been used in undersampled compressed‐sensing reconstructions. For instance, the L1‐SPIRiT^45^ and L1‐ESPIRiT^46^ methods combine parallel imaging with compressed sensing using a wavelet‐based regularization term and have been used in applications such as pediatric body imaging achieving acceleration factors up to 6×,^47^ liver imaging with ~5/6× acceleration,^48^ and ~5× accelerated cardiac 4D phase‐contrast imaging.^49^ Other approaches have used finite differences in the image (i.e. spatial TV) to constrain the accelerated reconstruction problem. For instance, 3D TV regularization has been used to accelerate 3D atrial LGE imaging up to 3.5×.^50^, ^51^

**FIGURE 5:**
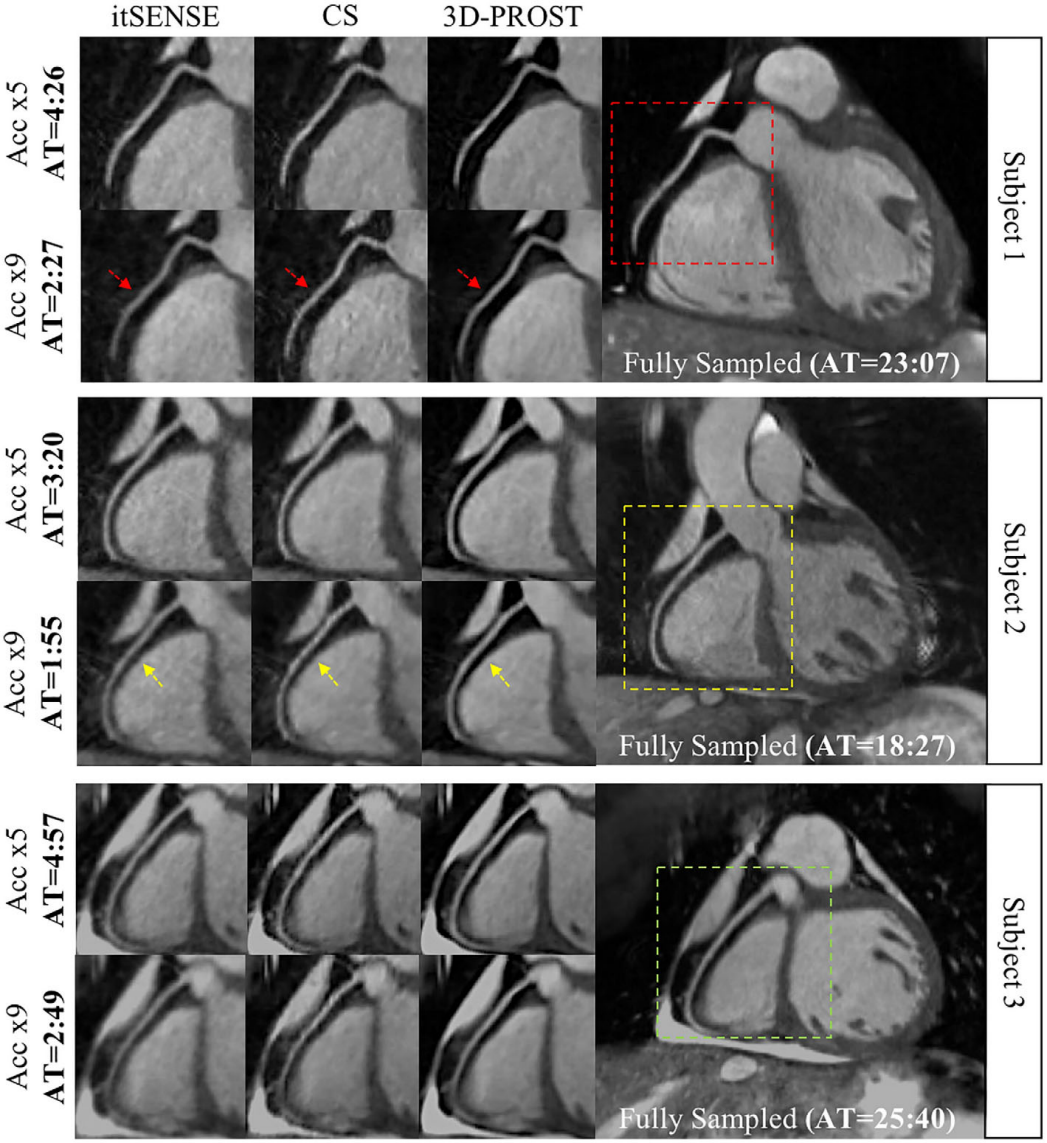
Comparison between conventional parallel imaging reconstruction (itSENSE), wavelet‐based compressed sensing (CS) and patch‐based low rank regularized (3D‐PROST) coronary MR angiography. 3D‐PROST enables good quality depiction of the coronary anatomy from highly accelerated scans. *Source*: Reproduced with permission from reference 56 Five‐minute whole‐heart coronary MRA with sub‐millimeter isotropic resolution, 100% respiratory scan efficiency, and 3D‐PROST reconstruction.

Both wavelet‐ and TV‐based regularization operate over the MR image as a whole and seek to exploit the intrinsic structure of the images. However, using a preselected transform may result in residual artifacts when the transform does not produce a sufficiently sparse representation. This may be the case in applications such as cardiac imaging, where there is a large interpatient variety in anatomy and signal‐to‐noise ratio levels, or if data acquisition is affected by motion. Alternative approaches, such as low‐dimensional‐structure self‐learning and thresholding (LOST)^52^ use local anatomical information in the form of patches learned from each dataset to create a patient‐dependent sparse representation. While LOST adds computational complexity compared to conventional compressed sensing, it has been shown to reduce image blurring in applications such as 3–5× accelerated 3D isotropic LGE imaging,^53^, ^54^ and 6× accelerated coronary MR angiography.^55^ This patch‐based approach has also been used in the context of low‐rank matrix representations. Methods such as PROST (3D patch‐based undersampled reconstruction)^56^ exploit local (i.e. within patches) and nonlocal (i.e. between similar patches) similarities (Fig. 4, bottom) and have been shown to produce good quality images from highly undersampled coronary MR angiography (Fig. 5)^57^, ^58^ and other applications.

## Exploiting Relaxation/Parametric Redundancies

Quantitative imaging of MR tissue parameters, such as T_1_, T_2_, or T_2_* mapping, is a promising approach for objective and reproducible tissue characterization and has shown promise for increased diagnosis sensitivity and specificity in a variety of clinical applications. Conventional techniques for parameter mapping acquire a sequence of images with varying contrast weighting, which are subsequently used to fit known physical models of the signal behavior. For example, in conventional T_1_ mapping, either saturation recovery or inversion recovery preparation pulses are used to produce several T_1_‐weighted images, which are typically reconstructed separately. Then, for each pixel in the image, a fitting to a two‐ or three‐parameter model of the T_1_ recovery curve is used to retrieve a T_1_ value. Such an approach results in long scan times, because it requires the acquisition of multiple contrasts to produce a good quality fit. Thus, many developments in image acceleration have focused on accelerating the acquisition of parameter maps.

Model‐based reconstruction approaches have been used to directly reconstruct parametric maps from undersampled *k*‐space data, by incorporating a known analytical signal model into the forward model of the acquisition. Combined with multichannel acquisitions and spatial regularization, these methods have shown to enable accelerated T_1_ ^59^, ^60^ and T_2_ ^61^, ^62^, ^63^ mapping with moderate undersampling factors. The known signal model has also been used to fill the undersampled k‐space, improving final image quality of the contrast‐weighted images, as proposed by Tran‐Gia et al^64^ for T_1_ mapping. This approach uses an iterative algorithm that alternates between using the current estimate of the weighted images to fit a two‐parameter model, and then using the resulting maps and the known signal model to predict how the fully sampled *k*‐space should look like for each contrast‐weighted image. Before the next iteration, the originally acquired lines of *k*‐space are re‐inserted into the predicted *k*‐space, ensuring data consistency. While originally applied to brain imaging,^64^, ^65^ this approach has been recently extended to cardiac T_1_ mapping.^66^

**FIGURE 6:**
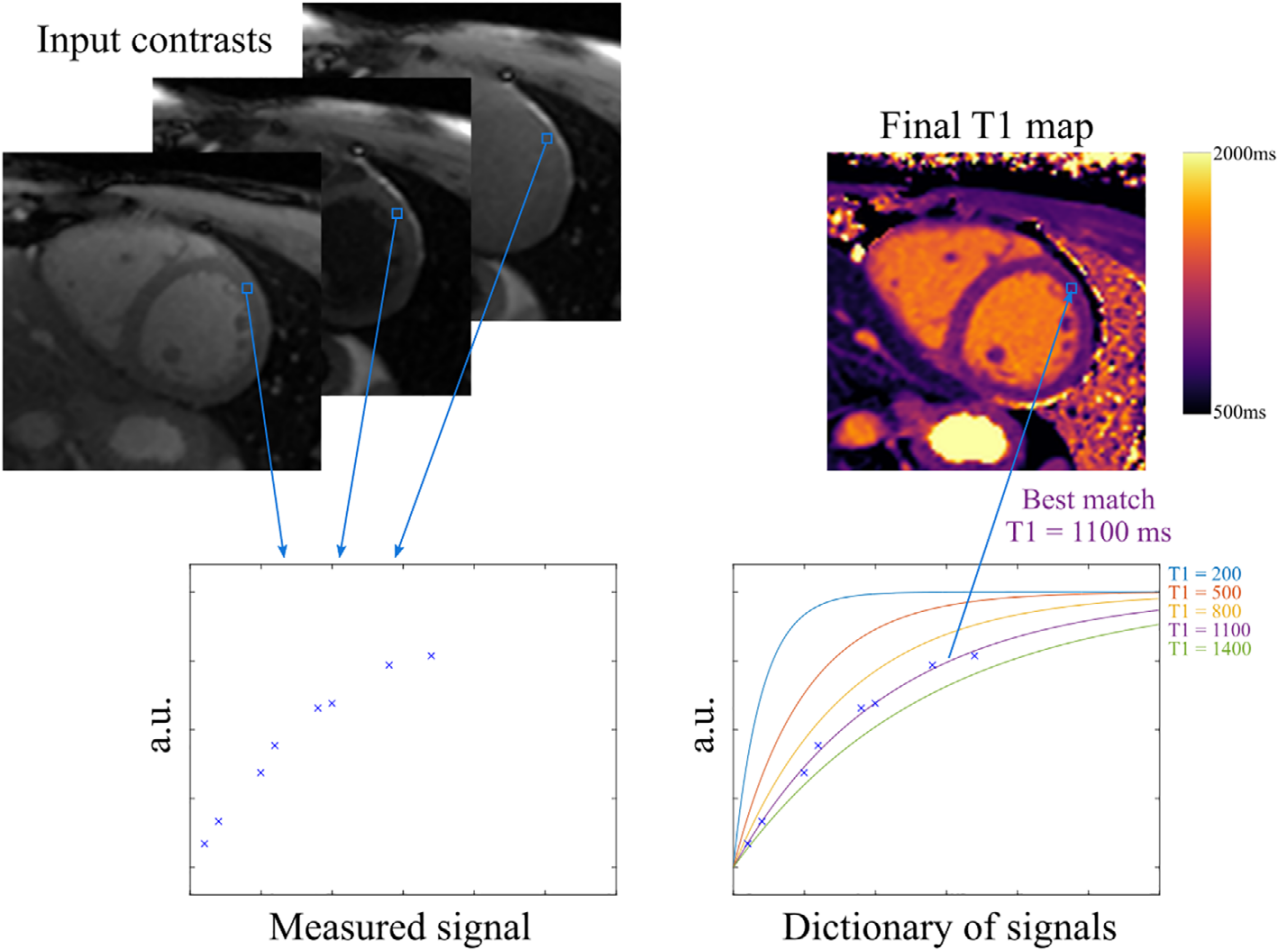
Illustration of dictionary‐based T1 quantitative mapping. Using knowledge of the signal model, a dictionary of potential signals can be created (bottom right). Then, this dictionary can be used to find the best match to the actual measured signal (bottom left) and retrieve the corresponding parameter value to produce the final T1 map (top right).

Alternatively, the knowledge of the signal model can be leveraged to create a *dictionary* of possible time signal prototypes, that is, a dictionary that relates a given parameter value to the series of pixel values that such parameter produces for a given acquisition sequence. In dictionary‐based quantitative mapping, this dictionary can be used to retrieve the parameter value for each voxel (Fig. 6). This dictionary can also be used as sparsifying transform in a compressed‐sensing framework, as demonstrated in the literature^6^ for T_1_ and T_2_ mapping of the brain and in the literature^67^ for cardiac T_1_ mapping. The dictionary of signals can also be used to linearize the parameter‐fitting problem in model‐based reconstruction, as demonstrated in the literature^68^ for highly accelerated (up to 16×) brain and abdominal T_1_ mapping.

While model‐based approaches can produce good results, they rely on the accuracy of the selected signal model to represent the MR pulse sequence used to acquire the data. Model‐agnostic methods have been developed to exploit the redundancy between contrasts in parameter mapping without requiring prior knowledge of the signal model. Velikina et al^69^ suggest that the signal evolution through the weighted images is usually smooth, and therefore the first‐ and second‐order derivative across contrasts is sparse. Using the L_1_ norm of the second derivative to regularize the reconstruction, 10× accelerated brain T_1_ mapping with good image quality was achieved.

**FIGURE 7:**
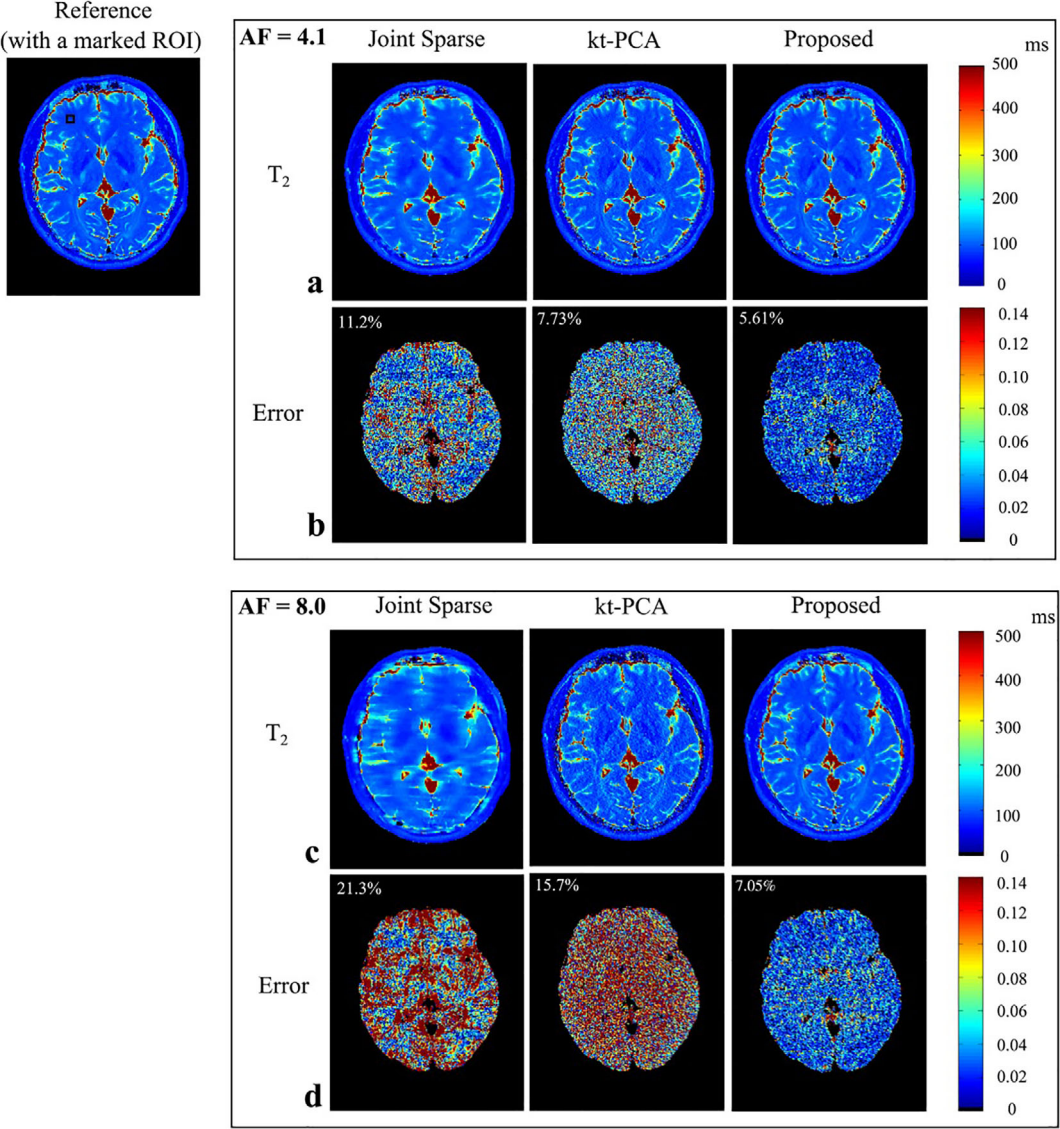
T_2_ maps of the human brain and associated errors for different acceleration factors (AFs). The authors propose a method that exploits both sparsity and low‐rank properties of multicontrast acquisitions (right column), showing an increased accuracy compared with methods that use only sparsity (Joint Sparse) or only low‐rank (kt‐PCA) constraints. *Source*: Reproduced with permission from reference 71

Alternatively, the redundancy of the images in the parameter direction can be exploited through low‐rank constraints. Petzschner et al^70^ propose that the dynamics along the changing contrast in parameter mapping can be described by a very small number of basis functions. Using low‐resolution training data, the proposed method uses PCA to estimate these basis functions and constrain the reconstruction, enabling 8× accelerated simultaneous T_1_ and T_2_ mapping of the brain. A different approach is to use the low‐rank constraint directly, by rearranging the acquired images into a space‐parameter matrix, where each column represents a contrast weighting. The resulting matrix, also known as the Casorati matrix, is low rank, and can be used to recover good quality images from undersampled data. This approach is used in the literature,^71^ together with a finite difference sparsity constraint across contrasts, to achieve up to 8× accelerated T_2_ mapping and 5× accelerated T_1_ mapping of the brain, with improved accuracy compared with methods that use only sparsity or only low‐rank constraints (Fig. 7). The low rankness of the images can be further exploited if the Casorati matrix contains in each column local patches of the images (i.e. using a locally low‐rank constraint), as demonstrated in the literature.^72^ Authors show that local low rank produces superior images than global low rank for multiecho spin‐echo T_2_ mapping, while comparable results between both approaches are obtained for variable flip angle T_1_ mapping.

## Exploiting Spatiotemporal Redundancies

**FIGURE 8:**
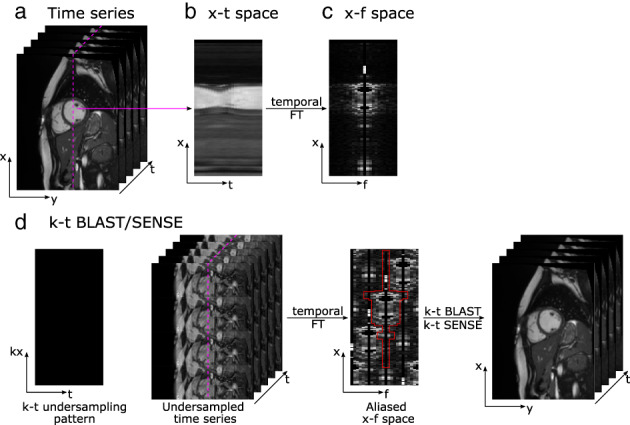
Example of spatiotemporal acceleration in cardiac cine images. (a) Example time series of dynamic cardiac images represented as a stack of images. Taking a column from each frame (white dotted line) results in the x‐t space representation (b). (c) When taking the Fourier transform in the temporal direction, a compact *x*‐*f* space is obtained (*x* and *f* denote spatial position and temporal frequency, respectively). (d) One alternative for spatiotemporal acceleration is the *k*‐*t* BLAST/SENSE method, shown here with 4x acceleration alongside corresponding undersampled time series images. By using low‐resolution training data, the fold‐over artifacts in the x‐f space produced by the undersampled acquisition can be filtered out (red lines) and unaliased images can be reconstructed.

Before the introduction of compressed sensing and the idea of sparse representations in the mid‐to‐late 2000s, image acceleration methods focused on approaches to remove the aliasing produced by undersampling k‐space using regular sampling patterns. In the context of dynamic MRI, pioneer methods simultaneously exploited spatial and temporal redundancies to push the boundaries of accelerated data acquisition. By arranging the acquired data into an extended *k*‐space with an additional time axis *t*, the so‐called *k*‐*t* approaches exploit the compact structure of the *x*‐*f* space (where *x* denotes the spatial dimension and *f* is the temporal frequency) in applications with periodic motion that affects only a portion of the image (Fig. 8).

UNFOLD^7^ and k‐t BLAST/SENSE^8^ were some of the first methods to exploit joint spatiotemporal redundancies in dynamic MRI. By using a lattice‐sampling pattern in *k*‐*t* space, these methods aim to produce an aliased *x*‐*f* space with little overlap between replicas, enabling the recovery of the original un‐aliased *k*‐*t* space by simple filtering. In combination with parallel imaging (i.e., using the *k*‐*t* SENSE method), this approach enabled up to 4–5× acceleration for applications such as 3D cardiac cine and 2D phase‐contrast flow imaging.^73^, ^74^

*k*‐*t* BLAST/SENSE produces good‐quality images when the motion of the acquired data is periodic and smooth. However, when motion is nonperiodic or when the contrast of the images is changing throughout the acquisition, such as the case in perfusion cardiac imaging, the *x*‐*f* space is less compact and more sophisticated techniques are required to resolve the undersampling artifacts. For instance, SPEAR improved on this method by including the acquisition of some fully sampled frames for increased temporal resolution.^75^ *k*‐*t* PCA^76^ extended the k‐t BLAST/SENSE method by unaliasing the signals in the x‐PC (principal component) space instead of the *x*‐*f* space. Authors show that using PCA in the temporal direction enables a more compact representation, allowing for increased undersampling rates compared to the conventional k‐t BLAST/SENSE and achieving over 6× net acceleration in myocardial perfusion imaging.

*k*‐*t* methods remained popular after the introduction of compressed sensing. Instead of the lattice sampling pattern described earlier, these novel methods used pseudo‐random acquisition patterns in the *k*‐*t* space to produce noise‐like artifacts that could then be removed using a combination of temporal and spatial sparsity constraints. *k*‐*t* FOCUSS^77^, ^78^ uses a prediction approach to produce a first estimate of all the images in the time series, and then uses the residual between the reconstructed image and this prediction as sparsifying transform. This method has been applied to both Cartesian and radial trajectories, achieving 6× accelerated cardiac cine imaging and 4× accelerated cardiac MR tagged imaging with good image quality.^79^ As mentioned in previous sections, *k*‐*t* SPARSE^22^ uses a wavelet transform in the spatial domain together with a FT along the temporal dimension to produce a sparse representation of dynamic cine MR images.

**FIGURE 9:**
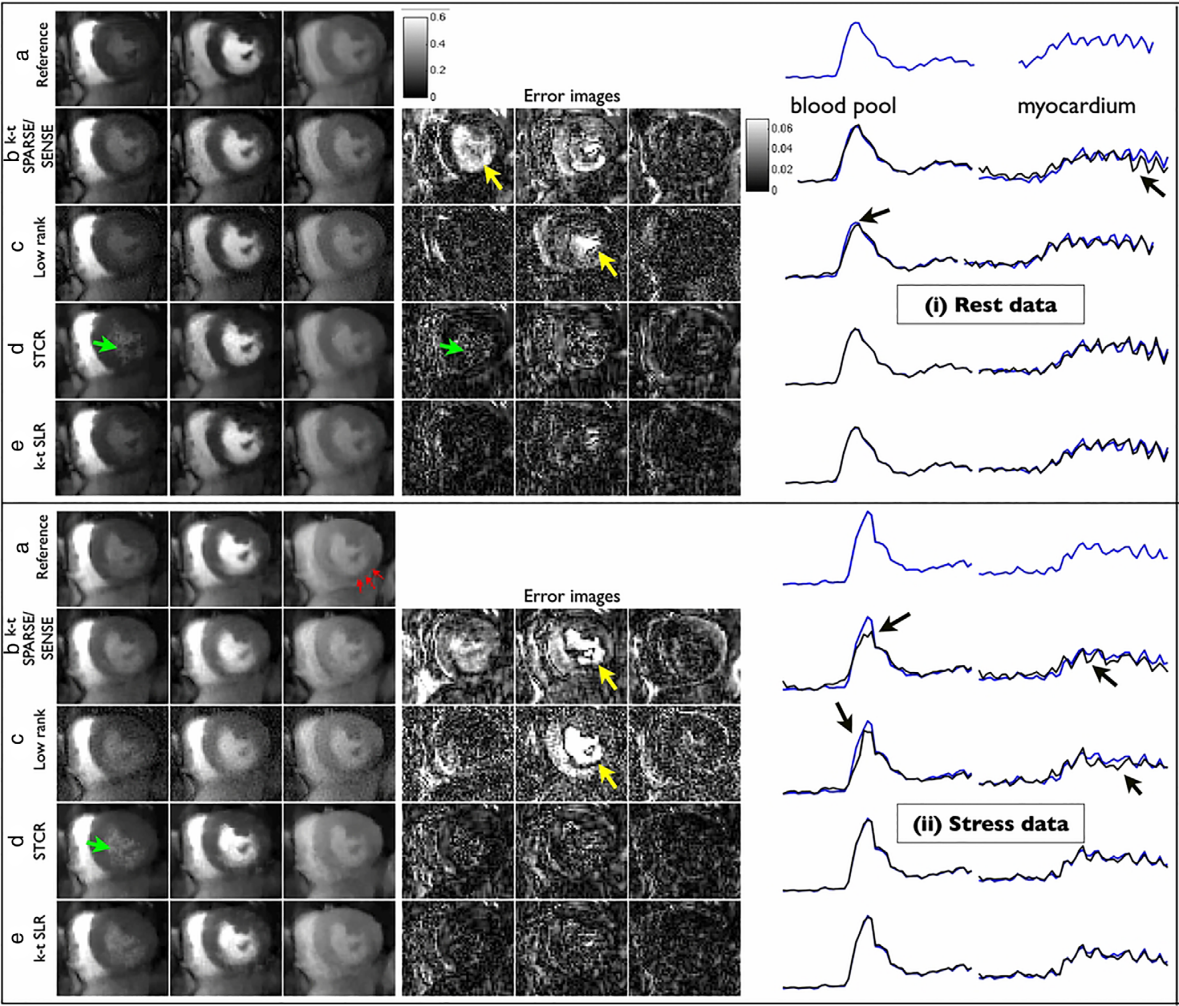
Accelerated cardiac perfusion images from a patient with myocardial ischemia. The first three columns correspond to peak right ventricular blood enhancement, transition between right and left ventricle, and peak myocardial wall enhancement. Error images and time curves corresponding to the regions of interest are also depicted. The authors compare their proposed low‐rank plus sparse reconstruction (*k* − *t* SLR) against reconstructions that use sparsity only (*k* − *t* SPARSE/SENSE), low rank only and a spatio‐temporal constrained reconstruction (STCR), observing that *k*‐t SLR produced better quality across frames, with less blurring (yellow arrows) or patchy artifacts (green arrows). *Source*: Reproduced with permission from reference 82

In order to further exploit spatiotemporal correlations, dynamic MR images can be rearranged into a space–time Casorati matrix, where each column represents a temporal frame. Methods such as k‐t SLR^80^ and *k*‐*t* partial separability^81^ aim to reconstruct dynamic MR images that are both sparse and low rank. *k*‐*t* SLR uses a combination of spatial wavelets and temporal FT to sparsify the images, together with a low‐rank constraint to enable highly accelerated (11×) cardiac perfusion. Compared to techniques that only use sparsity or only use low rank, authors showed that *k*‐*t* SLR improved definition and temporal fidelity in free‐breathing cardiac perfusion imaging^82^ (Fig. 9).

Alternatively, the acquired images can be decomposed into a low‐rank component plus a sparse component, as proposed by Otazo et al.^83^ In dynamic MR imaging, the unchanging or slowly changing background can be modeled as the low‐rank component, while the sparse component might contain the information of moving objects or structures with sudden changes in contrast. The low‐rank plus sparse approach was first demonstrated in cardiac cine and cardiac perfusion imaging, with an 8× accelerated acquisition, and in 48× accelerated abdominal DCE‐MRI, showing promise for dynamic imaging with sub‐second temporal resolution.

Dictionary learning‐based techniques have also been used to produce spatiotemporally sparse representations and thus further accelerate dynamic imaging. Instead of relying on physical data models to create the dictionaries (such as is the case for parametric mapping described above), these techniques learn the dictionary from the data itself, producing highly adaptable image‐specific sparse transformations. While the computation of this patient‐specific dictionary adds some overhead to the image reconstruction process, dictionary‐based techniques have been shown to outperform conventional compressed sensing approaches by producing images with superior temporal fidelity, particularly at high acceleration factors. A dictionary^84^ of temporal signals is used to achieve 7.5× accelerated cardiac perfusion imaging, while up to 16× accelerated cardiac cine is demonstrated in the literature,^85^, ^86^ using a dictionary of spatiotemporal patches.

## Exploiting Multiple Dimensions: Beyond Spatiotemporal

In most of the approaches discussed so far, the aim of the MR imaging process is to capture the behavior of the signal in a single dynamic dimension. Many of the *k*‐*t* methods described above focused on efficient cardiac cine imaging, for example, while methods exploiting parametric redundancy focused on obtaining a single parameter map with as few *k*‐space samples as possible. In most of these imaging tasks (i.e. cardiac cine, parameter mapping), there are other dynamic dimensions in play during the data acquisition process. For instance, cardiac imaging and abdominal imaging are affected by respiratory motion, and in most of the previously described approaches, such effect is minimized or mitigated through respiratory gating, breath holding and/or motion binning. An alternative approach is to incorporate more dynamic dimensions into the reconstruction problem by considering them as and additional dimensions of a multidimensional imaging task.

High‐dimensional MR imaging approaches have been developed to improve efficiency in the MR acquisition process to capture data correlation in multiple dimensions simultaneously, beyond just spatiotemporal or spatio‐spectral redundancies. For example, 5D flow^87^ acquires data continuously for 4 minutes, without ECG triggering and under free breathing, using a pseudo‐radial Cartesian trajectory. The acquired data are then sorted into four respiratory states, cardiac phases and seven velocity encodings, resulting in a ~19× acceleration factor. Using a locally low‐rank constraint to exploit cardiac and respiratory redundancies, good quality respiratory motion‐resolved 4D flow images are obtained.

In XD flow^88^ a similar approach is used, with data acquired with a variable‐density Cartesian radial view‐ordering trajectory after the injection of a contrast agent, adding an additional dimension (dynamic contrast enhancement) to the reconstruction problem. However, by projecting this seven‐dimensional dataset into a smaller number of dimensions, authors can reconstruct images that highlight different subspaces of the data, including respiratory‐resolved 4D flow imaging, perfusion imaging and respiratory and cardiac function, providing for a comprehensive imaging study from a single examination. To achieve this, a compressed‐sensing approach is used, with a wavelet transform to promote sparsity in the spatial dimension, and finite differences in all the other dimensions (cardiac, respiratory, contrast enhancement).

**FIGURE 10:**
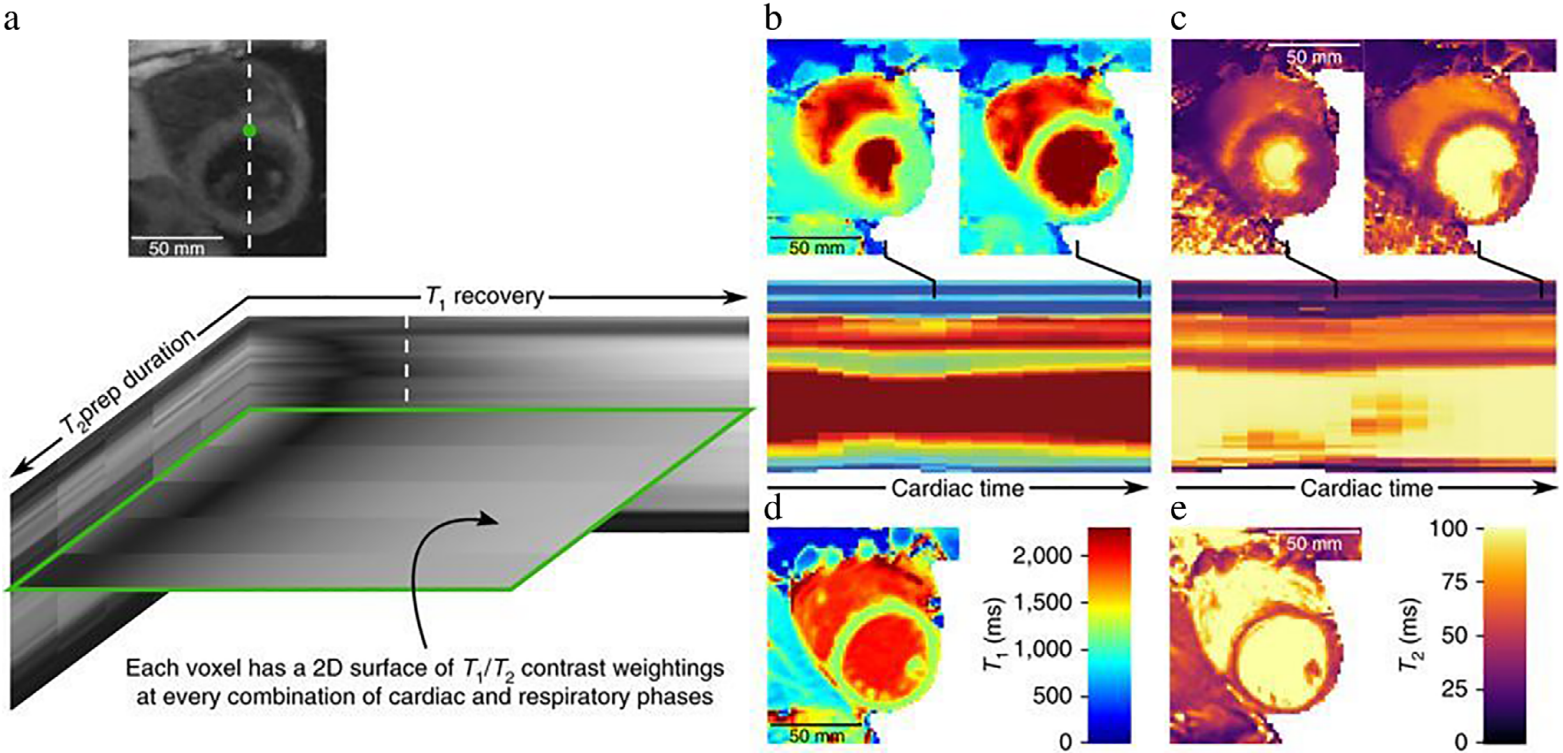
MR Multitasking approach for non‐ECG, free‐breathing joint T1/T2 mapping in the myocardium, compared with standard T1 MOLLI (d) and T2prep bSSFP (e) mapping. Multitasking yields contrast variation along the T1 recovery and T2prep duration dimensions (a), producing T_1_ and T_2_ maps not only are co‐registered but are also cardiac resolved (b, c), so that T1 and T2 maps can be obtained at each cardiac phase. *Source*: Adapted by permission from reference 9.

MRI multitasking^9^ uses a different approach for accelerated multidimensional imaging. Using a low‐rank tensor decomposition instead of the conventional low‐rank matrix formulation, the multitasking approach exploits the correlation between all dimensions present in the acquired data (eg spatial, contrast, and cardiac and respiratory motion dimensions) (Fig. 10). As other low‐rank models, the multitasking approach does not require to predefine a transformation to enforce sparsity, instead learning a compact representation from the data itself. Multitasking represents multidimensional MR images with N dynamic dimensions as a (*N* + 1)‐way tensor A and employs Tucker decomposition^89^ to model A as the product of a core tensor G and (*N* + 1) factor matrices:

(6) $$A=G\times_{1}U_{x}\times_{2}U_{t1}\times_{3}U_{t2}\cdots \times_{N+1}U_{\mathrm{tN}}$$

where $\times_{i}$ is the *i*th mode product, $U_{x}$ contains spatial basis functions, and $U_{\mathrm{ti}}$ contains basis functions for the dynamic dimension $t_{i}$. Given the high dimensionality of the data, a full Tucker decomposition becomes computationally prohibitive. In order to reduce computational burden, multitasking proposes to preestimate basis functions for each dynamic process from subject‐specific pilot data, obtaining an estimate for $\Phi =G\times_{2}U_{t1}\times_{3}U_{t2}\cdots \times_{N+1}U_{\mathrm{tN}}$. Thus, the tensor decomposition equation simplifies to $A=\Phi \times_{1}U_{x}$. Then, the multitasking image reconstruction process recovers the spatial basis functions $U_{x}$ solving

(7) $$\underset{U_{x}}{\text{argmin}}{\left\|A\left(\left[\mathrm{FC}U_{x}\right]\Phi \right)-y\right\|}_{2}^{2}+R\left(U_{x}\right)$$

where A is the sampling operator, F is the Fourier transform, C represents the coil sensitivity profiles, and $R\left(\right)$ is a regularization functional that can be used to enforce additional prior information about the image, such as sparsity. Multitasking was initially demonstrated for motion‐resolved cardiac T_1_ mapping, joint cardiac T_1_/T_2_ mapping and first‐pass myocardial perfusion from short scan of around 60 seconds,^9^, ^90^ and has since been applied for comprehensive aortic imaging,^91^ and quantitative abdominal DCE imaging,^10^ among others.

High‐order low‐rank tensor decomposition has also been used in the context of patch‐based reconstruction of undersampled multidimensional images. The HD‐PROST method^92^ arranges the multicontrast images as a patch‐based third‐order tensor in order to exploit local, nonlocal, and spectral (i.e. between contrasts) redundancies. This third‐order tensor is formed by the following steps: first, block matching is used to find the K most similar 3D patches in a local area of the multicontrast images; then the patches are unfolded together in a 2D matrix, and the tensor is finally formed by stacking the unfolded patches in the contrast dimension, resulting in a N×K×L tensor, with N is the number of elements in the patch, and L is the number of contrast‐weighted images. The reconstruction problem is then formulated as

(8) $$\underset{x}{\text{argmin}}{\left\|\mathrm{Ex}-y\right\|}_{2}^{2}+\sum_{p}\lambda_{p}{\left\|T_{p}\right\|}_{*}$$

where $T_{p}$ is the third‐order tensor centered at pixel p, ${\left\|\right\|}_{*}$ is the nuclear norm enforcing low rankness, and $\lambda_{p}$ is the regularization parameter. This approach has been applied to accelerated 3D multicontrast whole‐heart imaging, including T_1*ρ*_ mapping,^93^ T_2_ mapping,^94^ water/fat joint T_1_/T_2_ mapping,^95^ and water/fat LGE imaging.^96^ By extending the patch search to the cardiac dimension, this approach has also been used for accelerated 3D cardiac cine imaging.^97^

## Future Perspectives

Advances in the field of image acceleration and undersampled reconstruction methods that exploit sparsity and low‐rank representation have enabled multicontrast MR imaging with significant improvements in spatial and temporal resolution within clinically feasible scan times. Future clinical validation of these techniques is proposed to bear additional advantages. Accelerated multidimensional MR imaging approaches produce large, rich datasets, with extra contrasts/dynamics compared to conventional MR imaging, currently not fully explored in the clinical setting. Some of these dimensions, for instance, the respiratory dimension in respiratory‐resolved coronary MR angiography^32^, ^33^ or the cardiac and respiratory dimensions in multitasking quantitative cardiac parametric mapping,^9^, ^90^ may carry additional diagnostic information. These approaches also present an opportunity to include additional dynamic dimensions that have not been investigated so far, for instance, arrhythmia‐resolved imaging in patients with irregular heart rate.

In this review, we mostly focused on approaches that resolve the problem of physiological motion by reconstructing cardiac‐ or/and respiratory‐resolved images. There are, however, alternative concepts that are currently being investigated with intense research interest. Recently, proposed methods that estimate and compensate for intraframe physiological motion have been shown to improve performance and enable further acceleration.^98^, ^99^ These approaches have been successful in reducing artifacts arising from motion; however, they add further complexity to the image reconstruction problem and extend computation times. Furthermore, they require the tuning of additional parameters, for instance, for accurate motion estimation.

### Limitations

One of the limitations of the methods described in this review is their reliance on simplified models of the data acquisition and/or the physiology of the subjects, which is then exploited to reduce the amount of data required to produce good quality images. For instance, many of the introduced methods have assumed that the respiratory or cardiac cycles are periodic or quasi‐periodic and use surrogate signals to group data acquired over multiple cycles into quasi‐static states. However, variations in breathing patterns and changes in heart rhythm over the scan might result in increased artifacts in the reconstructed images due to the breach of this assumption. Or, for example, in accelerated quantitative T1 and T2 mapping, models that use simplified mono‐exponential functions overlook the effect of field inhomogeneities, magnetization transfer and/or in‐plane and through plane physiological motion, among others. The use of simplified models for image acceleration might in this case result in bias and imprecision in the resulting parametric maps and therefore restricts the acceleration that can be achieved.

Another major limitation of the more advanced approaches for multidimensional imaging described in this article is the increased complexity of the image reconstruction algorithms, and the associated increased computation time required to perform this task. Most of these approaches rely on off‐line reconstruction in powerful computers after image acquisition. Consequently, most clinically used MR imaging protocols still rely on parallel imaging and partial Fourier, and more recently compressed sensing, to reduce scan time, achieving moderate acceleration factors. Furthermore, many of the methods discussed in this article require the selection of one or more hyperparameters that control the level of image regularization. Careful tuning of such parameters is required to obtain images with sufficient removal of undersampling artifacts without resulting in blurring and loss of detail in small features.

Both limitations might be addressed in future by deep learning‐based image reconstruction approaches that can reduce image reconstruction time to a few seconds.^100^ As described in equation (1), conventional accelerated MR image reconstruction techniques aim to recover an image from undersampled *k*‐space data. Information about the data acquisition process is incorporated into the forward operator, while prior information about the scanned object can be used to regularize the problem, that is, to constrain the search for an optimal image. In contrast, deep‐learning image reconstruction methods aim to learn a mapping function (i.e. a neural network) from large sets of data, through an upfront learning process known as training. After training, this learned mapping can be applied to unseen undersampled data to produce good‐quality images in a fraction of the time required by conventional iterative image reconstruction algorithms. Deep‐learning reconstruction networks can receive as an input an undersampled image and/or *k*‐space data, and a variety of techniques have been proposed in the literature that differ in terms of how these data are processed and whether information such as the one contained in the forward operator are required by the network. A comprehensive review of such approaches is out of the scope of this article and can be found in the literature.^43^, ^100^, ^101^ While promising, current deep learning‐based techniques have focused primarily on single‐contrast MR imaging and more recently on multicontrast imaging of static organs such as the brain or the knee. Their applicability to multidimensional imaging in the presence of cardiac and respiratory motion, and where reference fully sampled reference datasets might not be available, is an emerging area and remains to be studied.

Most of the approaches described in this article have been tested in healthy subjects and compared against conventional techniques in single‐center studies with relatively small cohorts of patients. Moreover, direct comparison between approaches has been challenging due to the lack of availability of open data repositories that can be used as a benchmark for the different methods. Therefore, in most cases, there is no sufficient evidence to provide a single recommendation about which method is preferable for any given clinical application. To address this limitation, larger multicenter studies and open data repositories containing raw *k*‐space data are required to fully characterize and compare the performance of these methods in the presence of disease. Multidisciplinary collaboration between scientists, engineers and clinicians is crucial to translate decades of innovation to clinical impact. Leveraging advanced acceleration methods into clinical service will improve scan and cost efficiency, patients' experience and staff's workflow, in addition to enhancing image quality (fewer breath‐holds, reduced patient motion) and hence diagnostic confidence.

### Conclusion

Unfolding the dimensionality of MR data into comprehensive image acquisition and reconstruction strategies has been progressively offered ingenious and sophisticated solutions, with prospective clinical adoption. These approaches have the potential of paving the way toward highly efficient push button MR exams.
